# Early Aortic Autograft Infective Endocarditis with Perivalvular Abscess Following the Ross Procedure

**DOI:** 10.3390/jcm15020611

**Published:** 2026-01-12

**Authors:** Peter Snopek, Štefan Lukačín, Ingrid Schusterová, Adrián Kolesár, Jozef Hasilla, Milan Klačanský, Pavol Fülöp, Tibor Porubän, Štefan Tóth, Mariana Dvorožňáková

**Affiliations:** 1 Department of Cardiology, Faculty Hospital, 950 01 Nitra, Slovakia; 2 Faculty of Social Sciences and Health, University of Constantin the Philosopher, 949 01 Nitra, Slovakia; 3 Department of Heart Surgery, Faculty of Medicine, University of Pavol Jozef Šafárik and East Slovak Institute of Cardiovascular Diseases, 040 11 Košice, Slovakia; 4 Department of Imaging Techniques, Faculty of Medicine, University of Pavol Jozef Šafárik and East Slovak Institute of Cardiovascular Diseases, 040 11 Košice, Slovakia; 5 2nd Department of Cardiology, Faculty of Medicine, University of Pavol Jozef Šafárik and East Slovak Institute of Cardiovascular Diseases, 040 11 Košice, Slovakia; 6 1st Department of Cardiology, Faculty of Medicine, University of Pavol Jozef Šafárik and East Slovak Institute of Cardiovascular Diseases, 040 11 Košice, Slovakia; 7 Department of Gerontology and Geriatrics, Faculty of Medicine, Pavol Jozef Šafárik University—University Hospital of St. Michael, Murgašova 1, 040 86 Košice, Slovakia

**Keywords:** ross procedure, infective endocarditis, echocardiography, antibiotics

## Abstract

**Background**: The Ross procedure provides excellent outcomes in young adults requiring aortic valve replacement, with lower rates of infective endocarditis (IE) compared to prosthetic valves. Early postoperative IE of the autograft is exceptionally rare, with only isolated cases reported in major registries. **Case Summary**: We report a 40-year-old man with bicuspid aortic valve and a history of two previous episodes of native valve endocarditis who underwent Ross procedure with Ozaki modification. Ten days postoperatively, he developed fever and was diagnosed with early autograft IE with perivalvular abscess formation. Despite negative blood cultures (due to prior antibiotic administration), clinical findings met modified Duke criteria for possible IE. Imaging revealed perivalvular abscess with subsequent pseudoaneurysm development, while the autograft leaflets remained functional. The patient was successfully treated with 6-week combination antibiotic therapy without requiring surgical reintervention. After one year of follow-up, he remains asymptomatic with stable pseudoaneurysm size and preserved valvular function. **Conclusions**: Early IE following Ross procedure, though rare, should be considered in patients presenting with postoperative fever. This case demonstrates that conservative management with appropriate antibiotic therapy can be successful even with perivalvular complications, provided there is hemodynamic stability and close multidisciplinary monitoring. Long-term surveillance remains essential given the persistent structural abnormalities.

## 1. Introduction and Clinical Significance

The Ross procedure, first described by Donald Ross in 1967 [1], represents a unique surgical technique for aortic valve replacement that involves transplanting the patient’s own pulmonary valve (autograft) into the aortic position, while the pulmonary valve is subsequently replaced with a pulmonary homograft. This procedure offers several distinct advantages, particularly in young adults, including superior hemodynamic performance, absence of anticoagulation requirements, and the potential for autograft growth in pediatric patients [2,3]. The autograft’s living tissue characteristics provide excellent durability and resistance to infection compared to mechanical or bioprosthetic valves. Various modifications of the technique exist, including the Ozaki modification [4], which involves aortic valve reconstruction using autologous pericardium for certain cases of aortic stenosis.

Despite its advantages, the Ross procedure is technically demanding and associated with several potential complications. Early postoperative complications include bleeding, cardiac arrhythmias, and ventricular dysfunction. The most significant long-term concerns involve autograft-related complications, including progressive autograft dilation leading to aortic regurgitation, which may require reintervention in 15–25% of patients within 20 years [3,5]. The pulmonary homograft can also develop stenosis or regurgitation over time, though typically at a slower rate than the autograft complications [6]. Structural valve deterioration, particularly in the autograft position, represents a major challenge in long-term follow-up. Additional risks include thromboembolic events and endocarditis.

IE following the Ross procedure, while less common than with prosthetic valves [7], demonstrates a distinct pattern: it predominantly affects the pulmonary homograft in the right ventricular outflow tract, with reported incidence rates of 1–3% in long-term follow-up [5]. In contrast, early postoperative IE of the aortic autograft is exceptionally rare, with only isolated cases reported in major international registries [8,9]. This rarity may reflect what observational registry data suggest is relatively lower susceptibility to infection of living autologous tissue compared to non-viable homograft material. When autograft endocarditis does occur, it typically presents later in the postoperative course rather than in the early postoperative period. Perivalvular complications, such as pseudoaneurysm formation and abscess development, represent serious sequelae when they occur with autograft IE. Aortic regurgitation, a critical complication that may develop progressively, requires multimodality imaging assessment for optimal management [10].

We present a rare case of a 40-year-old man with a history of recurrent native valve endocarditis who developed early aortic autograft infective endocarditis with perivalvular abscess following the Ross procedure with Ozaki modification [4], successfully managed with conservative antibiotic therapy. This case highlights the unusual presentation of early autograft endocarditis and emphasizes the importance of comprehensive multimodality imaging in diagnosis and management.

## 2. Case Presentation

We present a 40-year-old man with a bicuspid aortic valve and a history of aortic valve infective endocarditis (IE) in December 2015 caused by *Staphylococcus warneri*. In March 2016, he developed recurrent aortic valve IE caused by *Aerococcus viridans*, which was complicated by amaurosis fugax in the left eye and retinal hemorrhage in the right eye. The repeated episodes of aortic valve endocarditis managed conservatively resulted in progressive aortic regurgitation that eventually became severe. After consultation between the patient, cardiologist, and cardiac surgeon, aortic valve replacement was planned using the Ross procedure with the Ozaki modification. The operation was performed on 17 January 2025, at the East Slovak Institute of Cardiovascular Diseases in Košice, Slovakia. The immediate postoperative course was uneventful, and the patient was discharged in stable condition for outpatient follow-up.

On 27 January 2025, the patient developed fever, and his general practitioner initiated empiric antibiotic treatment with amoxicillin-clavulanate 750 mg twice daily. Despite antibiotic therapy for three days, the fever persisted. He was subsequently examined by a district cardiologist who suspected IE and referred him for hospitalization at the Cardiology Department of Nitra Faculty Hospital on 31 January 2025.

**Figure 1:**
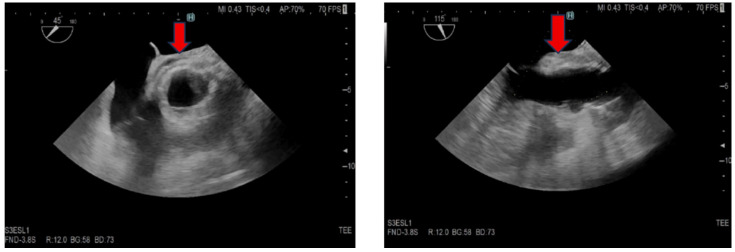
**Transesophageal echocardiography, short and long axis of the aorta.** The red arrow indicates thickened tissue in the aortic root area, suggestive of an abscess.

Upon admission, laboratory investigations revealed elevated inflammatory parameters and leukocyte count (detailed in Table 1). ECG and chest X-ray showed no significant abnormalities, with no evidence of pulmonary changes. Transesophageal echocardiography demonstrated tissue thickening in the aortic root (Figure 1), while the aortic valve appeared normal without regurgitation or stenosis. Blood cultures for aerobes and anaerobes were collected at 30 min intervals, and empiric treatment for early autograft endocarditis was initiated according to current ESC guidelines [11], consisting of vancomycin, gentamicin, and rifampicin.

**Figure 2:**
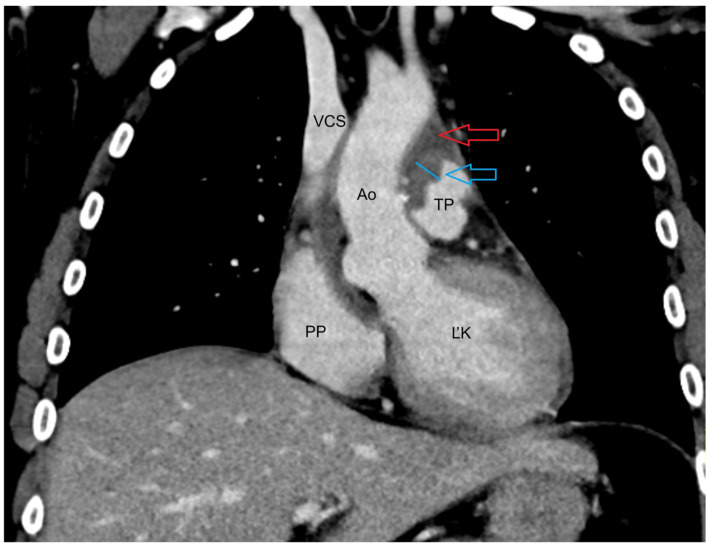
**CT of the upper chest venous phase transverse plane:** Blue arrow—fluid collection paraaortically to retrosternal with saturating wall, Red arrow—small air collection retrosternal in the fluid collection, Ao—aorta, TP—pulmonary trunk, VCS—superior vena cava, PP—right atrium, ĽK—left ventricule.

Given the suspected diagnosis of IE, we consulted with cardiac surgery, who initially recommended conservative management. Chest CT imaging (Figure 2) revealed an encapsulated effusion in the anterior mediastinum extending retrosternally, with an associated air bubble. Repeat transesophageal echocardiography after 7 days showed no significant interval changes. However, on 11 February, transthoracic echocardiography identified a pulsatile pseudoaneurysm with blood flow in the aortic root adjacent to the non-coronary and left coronary sinuses of Valsalva. The patient was subsequently transferred to the Department of Cardiac Surgery at the East Slovak Institute of Cardiovascular Diseases in Košice.

**Figure 3:**
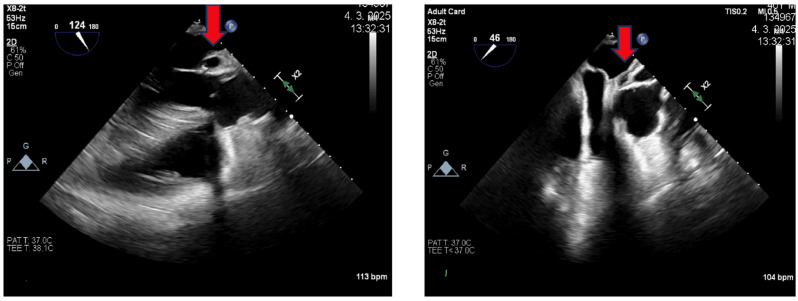
**Transesophageal echocardiography:** Long and short axis projection of the aorta. The red arrow shows a pseudoaneurysm.

Follow-up laboratory investigations demonstrated decreasing inflammatory parameters, and serial echocardiographic examinations confirmed a paravalvular pseudoaneurysm (21 × 38 mm) adjacent to the left and non-coronary sinuses of Valsalva (Figure 3). The lesion was confined to the aortic root without extension into the ascending aorta. Crucially, the pseudoaneurysm did not affect autograft valve function or cause hemodynamic compromise, supporting the decision for conservative antibiotic management rather than urgent surgical reintervention.

Following multidisciplinary consultation with cardiac surgery, conservative management was continued based on key favorable criteria: (1) hemodynamic stability with preserved cardiac function; (2) no progression of aortic regurgitation—the autograft maintained normal function with only mild regurgitation unchanged from baseline; (3) excellent clinical response to antibiotics with defervescence and declining inflammatory markers (C-reactive protein 7.4 mg/L); (4) negative repeat blood cultures; (5) absence of embolic events; and (6) localized abscess without ascending aortic or pulmonary valve involvement. Serial imaging showed partial abscess drainage, and the persistent pseudoaneurysm remained confined to the aortic root with flow toward the left ventricular outflow tract (LVOT). The surgical team agreed that given clinical stability and microbiological control, the substantial risks of reoperation in an infected field outweighed potential benefits. Pre-discharge PET-CT confirmed complete resolution of inflammation (no 18F-FDG uptake), validating the conservative strategy. The pulmonary valve and right ventricular outflow tract (RVOT) remained unaffected. The patient was discharged for outpatient surveillance.

**Figure 4:**
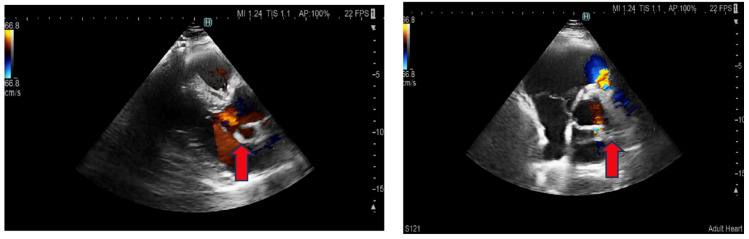
**Transthoracic echocardiography:** Parasternal long and short axis. The red arrow shows a pseudoaneurysm.

The patient has undergone regular clinical and echocardiographic monitoring since discharge. He has remained afebrile and asymptomatic for the past year. The pseudoaneurysm has remained stable in size (Figure 4), and both the aortic and pulmonary valves continue to demonstrate normal structure and function without regurgitation or stenosis.

## 3. Discussion

We present a rare case of early autograft infective endocarditis with perivalvular abscess formation following the Ross procedure with Ozaki modification in a 40-year-old patient with a history of recurrent native valve endocarditis. Despite the severity of the complication, occurring just 10 days postoperatively, the patient was successfully managed with conservative antibiotic therapy alone, without requiring surgical reintervention.

### 3.1. Uniqueness of the Case

This case is exceptional for two critical reasons: the remarkably early timing of infection onset (10 days postoperatively) and the successful conservative management despite significant perivalvular complications. Early prosthetic valve endocarditis occurring within 30 days of surgery is typically associated with perioperative contamination and carries a poor prognosis, particularly when complicated by perivalvular abscess formation. In the context of the Ross procedure, where autograft endocarditis is already exceptionally rare, the occurrence of IE within the first two weeks represents an almost unprecedented clinical scenario. The patient’s unique history of two prior episodes of native valve endocarditis (*Staphylococcus warneri* in 2015 and *Aerococcus viridans* in 2016) further compounds the rarity, as Ross procedures are typically performed in patients without such infectious backgrounds. The decision to proceed with a Ross procedure rather than a prosthetic valve in this high-risk infectious setting was based on observational data suggesting relatively lower rates of infection of living autologous tissue compared to mechanical or bioprosthetic alternatives. Despite developing the very complication the procedure aims to prevent, the autograft’s inherent biological properties—preserved endothelium and viable tissue—ultimately enabled successful medical management without surgical reintervention, validating the rationale for this challenging surgical choice [12].

### 3.2. Comparison with Existing Literature

The rarity of our case is supported by extensive registry data. The German-Dutch Ross Registry, which includes data from 12 cardiac surgery departments since 1988, reported only four cases of early endocarditis during the entire follow-up period among 1620 patients [8]. Late endocarditis was observed in 38 patients (2.3%; incidence rate 0.38 per patient-year), with only 18 autograft reoperations attributable to endocarditis (1.1%; incidence rate 0.18% per patient-year). Similarly, the European Ross Registry, including 2444 adults followed for a median of 9.2 years, documented autograft endocarditis leading to reoperation in only 37 patients (0.16% per patient-year) [9].

The rarity of our case is underscored by extensive registry data that specifically distinguish between early and late endocarditis. The German-Dutch Ross Registry [8] documented only four cases of early endocarditis among 1620 patients—representing just 0.25% of all Ross procedures. In stark contrast, late endocarditis was observed in 38 patients (2.3%), demonstrating approximately a tenfold higher frequency. This marked disparity confirms that endocarditis after Ross procedure typically occurs years rather than days or weeks after surgery. Of the autograft reoperations attributable to endocarditis, 18 cases (1.1%; 0.18% per patient-year) were performed, predominantly for late presentations. Similarly, the European Ross Registry, including 2444 adults followed for a median of 9.2 years, documented autograft endocarditis leading to reoperation in only 37 patients (0.16% per patient-year), with the vast majority occurring in the late postoperative period [9]. This temporal distribution—with late endocarditis being far more common than early infection—further emphasizes the exceptional nature of our case presenting just 10 days postoperatively.

The Toronto experience comparing Ross procedures with bioprosthetic aortic valve replacements between 1990 and 2014 confirmed the overall lower incidence of IE following the Ross procedure, but notably, even in this large series, early autograft endocarditis remained an extremely rare event [13]. A single-center study from Homburg, Germany, reviewing 30 consecutive autograft reinterventions between 1997 and 2022, identified IE as the indication for reoperation in only two cases [6], both presenting late in follow-up. The Rome experience with 108 adult Ross procedures reported no reoperations for aortic autograft endocarditis over their entire follow-up period, though 5 patients required intervention for pulmonary homograft endocarditis—all occurring late [14]. This consistent pattern across multiple registries—where autograft endocarditis is rare, and early presentation even rarer—positions our case as one of the earliest documented autograft infections in the published Ross procedure literature.

### 3.3. Benefits of Ross Procedure in Younger Patients

The selection of the Ross procedure for our patient, despite his concerning history of recurrent native valve endocarditis, warrants specific discussion as it represents a carefully considered decision in a high-risk infectious scenario. In young adults requiring aortic valve replacement, the choice is typically between mechanical prostheses requiring lifelong anticoagulation with associated hemorrhagic and thrombotic risks, bioprostheses with limited durability necessitating reoperation, or the Ross procedure. For our 40-year-old patient with two documented episodes of native valve IE, the risk of recurrent prosthetic valve endocarditis was particularly high—literature suggests that prior endocarditis increases the risk of prosthetic valve infection 3–5 fold compared to patients without such history [11]. The Ross procedure was therefore chosen specifically because the living autograft tissue offers superior resistance to infection compared to any prosthetic alternative. This advantage is supported by propensity-matched analyses showing lower IE rates with Ross procedures versus prosthetic valves [7], and is biologically explained by the autograft’s preserved endothelium and active immunologic defense mechanisms. The German-Dutch Ross Registry data, showing 10-year survival of 94.7% and freedom from autograft reoperation of 87.3–95.9% [8], further supported this choice. A network meta-analysis demonstrated that the Ross procedure achieves lower all-cause mortality compared to both mechanical (HR 0.58, *p* = 0.035) and bioprosthetic valves (HR 0.32, *p* < 0.001), with comparable reintervention rates while avoiding anticoagulation complications [15]. For this young, active patient with proven susceptibility to endocarditis, the Ross procedure represented the best long-term option—a decision that, despite the early postoperative complication, was ultimately validated by the successful conservative management enabled by the autograft’s biological properties.

### 3.4. Pathophysiology and Risk Factors

Several factors may have contributed to the development of early IE in our patient. First, the patient’s history of two previous episodes of native valve endocarditis suggests an underlying predisposition to endocardial infections, possibly related to immunological factors or persistent bacteremia sources. Second, the bicuspid aortic valve morphology, while not directly related to postoperative infection risk, indicates underlying connective tissue abnormalities that might affect healing processes.

The timing of infection onset (10 days postoperatively) falls within the critical early postoperative period where both perioperative contamination and early hematogenous seeding remain possible. The patient was discharged in stable condition on postoperative day 7 and developed fever only after returning home (day 10), which could suggest either delayed manifestation of perioperative inoculation or hematogenous spread from a community-acquired source. Unfortunately, the causative organism could not be identified despite multiple blood culture attempts. This culture-negative status resulted from empiric antibiotic therapy (amoxicillin-clavulanate) administered by the general practitioner for three days prior to hospital admission—a recognized challenge in IE diagnosis that substantially reduces the microbiological yield. Alternative diagnostic approaches for culture-negative endocarditis were considered, including serological testing for fastidious organisms (*Coxiella burnetii*, *Bartonella* species, *Brucella*), but were not pursued given the patient’s clinical response to empiric broad-spectrum therapy. Molecular methods such as 16S rRNA polymerase chain reaction on blood or tissue samples, which can identify pathogens even after antibiotic exposure, were not available at our institution at the time. Culture-negative infective endocarditis represents a recognized diagnostic challenge, accounting for 5–31% of IE cases depending on the series, and is most commonly caused by prior antibiotic exposure—precisely the scenario encountered in our patient. Despite negative cultures, the clinical presentation met modified Duke criteria for possible IE [16]: one major criterion (perivalvular abscess on imaging) and two minor criteria (fever and predisposing heart condition). The formation of a perivalvular abscess with subsequent pseudoaneurysm development indicates an aggressive local infectious process. Notably, the preservation of autograft leaflet function throughout suggests that the valve cusps themselves remained uninvolved, with infection primarily affecting the perivalvular tissues at the suture line—a pattern consistent with early perioperative contamination rather than hematogenous seeding of valve tissue.

### 3.5. Management Considerations

The successful conservative management of this case requires careful contextualization within current guidelines. According to 2023 ESC Guidelines for the management of endocarditis [11], surgery is generally indicated for IE with perivalvular complications (Class I recommendation). However, our case represents a carefully selected exception based on several convergent favorable factors: (1) hemodynamic stability with preserved cardiac function; (2) maintained autograft function with only trace aortic regurgitation; (3) rapid clinical response with defervescence and declining inflammatory markers; (4) negative surveillance cultures indicating microbiological control; (5) absence of embolic complications; (6) localized perivalvular involvement without extension; and (7) extremely high surgical risk of early reoperation (>20% mortality). PET-CT imaging confirmed complete resolution of inflammation. The six-week antibiotic course (vancomycin, gentamicin, rifampicin) achieved microbiological cure. Critically, this conservative approach should not be generalized—it applies only to exceptional cases with hemodynamic stability, preserved valve function, microbiological control, and close multidisciplinary monitoring with readily available surgical backup.

The persistent pseudoaneurysm despite successful infection eradication represents an ongoing challenge with uncertain long-term prognosis. Our surveillance strategy involves monthly clinical and echocardiographic assessments. Over 7 months, the pseudoaneurysm has remained stable with preserved autograft function. However, the patient understands that elective surgical intervention may ultimately be required. Thresholds for intervention include: pseudoaneurysm growth > 5 mm, new or worsening aortic regurgitation, hemodynamic compromise, or thrombus formation. This surveillance approach balances the risks of living with a structural abnormality against the substantial risks of reoperation in a young, currently asymptomatic patient.

### 3.6. Limitations and Lessons Learned

This case report has several limitations. The causative organism was not identified due to prior antibiotic therapy, limiting our understanding of the infection’s etiology and optimal targeted therapy. Long-term outcomes beyond one year remain unknown, and the eventual need for surgical intervention cannot be excluded.

Key lessons from this case include: (1) Early IE should be considered in any febrile illness following the Ross procedure, despite its rarity; (2) Conservative management with appropriate antibiotic therapy can be successful even in cases with perivalvular complications, provided there is close monitoring and absence of hemodynamic compromise; (3) A multidisciplinary approach involving cardiologists, cardiac surgeons, microbiologists, infectious disease specialists, and imaging specialists is essential for optimal management; (4) Long-term surveillance is mandatory given the structural complications that may persist despite microbiological cure.

### 3.7. Future Directions

Future directions in Ross procedure research focus on several key areas. Advances in imaging techniques, including three-dimensional echocardiography, cardiac magnetic resonance, and artificial intelligence-based analysis, promise earlier detection and more accurate assessment of autograft complications [10]. Novel surgical modifications, such as aortic root reinforcement techniques and personalized sizing strategies based on biomechanical modeling, aim to reduce the incidence of late autograft dilation [6]. Tissue engineering approaches investigating bioengineered heart valves may eventually provide alternatives to homografts in the pulmonary position. Additionally, ongoing registry studies and long-term outcome analyses continue to refine patient selection criteria and optimize timing of intervention for autograft dysfunction [2]. Understanding the molecular mechanisms underlying autograft adaptation and degeneration may lead to pharmacological strategies to prevent progressive dilation. Finally, the role of the Ross procedure in the era of transcatheter aortic valve replacement requires further investigation to define its optimal position in contemporary treatment algorithms for young patients with aortic valve disease.

## 4. Conclusions

This case demonstrates that early autograft endocarditis following the Ross procedure, while exceptionally rare, can be successfully managed with conservative antibiotic therapy when hemodynamic stability is maintained. The key clinical implications include: heightened awareness for infectious complications in patients presenting with postoperative fever, the feasibility of medical management in carefully selected cases with perivalvular involvement, and the critical importance of long-term echocardiographic surveillance given the unpredictable behavior of post-infectious pseudoaneurysms in this unique setting. This experience contributes valuable data to guide management decisions in similar rare presentations.

## Figures and Tables

**Table 1:** Results of laboratory tests upon admission to the cardiology department in Nitra.

Parameter	Value	Reference Range
Complete Blood Count		
Leukocytes	13.2 × 10^9^/L	4.0–10.0 × 10^9^/L
Erythrocytes	3.89 × 10^12^/L	4.5–5.5 × 10^12^/L
Hemoglobin	115.0 g/L	130–170 g/L
Hematocrit	0.350	0.40–0.50
Platelets	451 × 10^9^/L	150–400 × 10^9^/L
**Biochemistry**		
Urea	4.3 mmol/L	2.5–7.5 mmol/L
Creatinine	79.1 μmol/L	60–120 μmol/L
AST	0.30 μkat/L	0.17–0.85 μkat/L
ALT	0.64 μkat/L	0.17–0.83 μkat/L
GGT	1.17 μkat/L	0.15–1.10 μkat/L
ALP	1.69 μkat/L	0.67–2.15 μkat/L
CRP	49.72 mg/L	<5.0 mg/L
IL-6	139.0 pg/mL	<7.0 pg/mL
Procalcitonin	0.085 ng/mL	<0.05 ng/mL
**Electrolytes**		
Sodium	140.0 mmol/L	135–145 mmol/L
Potassium	4.71 mmol/L	3.5–5.1 mmol/L
Chloride	102.0 mmol/L	98–107 mmol/L

Abbreviations: AST—Aspartate Aminotransferase, ALT—Alanine Aminotransferase, GGT—Gamma-Glutamyl Transferase, ALP—Alkaline Phosphatase, CRP—C-Reactive Protein, IL-6—Interleukin-6.

## Data Availability

The original contributions presented in this study are included in this article. Further inquiries can be directed to the corresponding author.

## Abbreviations

The following abbreviations are used in this manuscript:

AS	Aortic Stenosis
AVR	Aortic Valve Replacement
CT	Computed Tomography
ESC	European Society of Cardiology
IE	Infective Endocarditis
LVOT	Left Ventricular Outflow Tract
RVOT	Right Ventricular Outflow Tract
